# Quantifying the population-level impact of expanded antibiotic treatment for cholera outbreak management

**DOI:** 10.1371/journal.pcbi.1013980

**Published:** 2026-02-18

**Authors:** Sharia M. Ahmed, Cormac R. LaPrete, Iza Ciglenecki, Andrew Azman, Daniel T. Leung, Lindsay T. Keegan

**Affiliations:** 1 Division of Epidemiology, University of Utah School of Medicine, Salt Lake City, Utah, United States of America; 2 Department of Epidemiology, Emory University, Atlanta, Georgia, United States of America; 3 Department of Mathematics, University of Utah College of Science, Salt Lake City, Utah, United States of America; 4 Médecins sans Frontières, Geneva, Switzerland; 5 Department of Epidemiology, Johns Hopkins Bloomberg School of Public Health, Baltimore, Maryland, United States of America; 6 Division of Infectious Diseases, University of Utah School of Medicine, Salt Lake City, Utah, United States of America; Fundação Getúlio Vargas: Fundacao Getulio Vargas, BRAZIL

## Abstract

Since 2021, there has been a resurgence in the number of cholera cases, countries affected, and the case fatality rate. Because most cholera patients recover without antibiotic treatment, current cholera treatment guidelines only consider the tradeoffs between patient recovery and antibiotic stewardship. However, antibiotics also greatly reduce bacteria shedding, creating a potential role for antibiotics in cholera outbreak response through reduced transmission. We developed a compartmental model of cholera transmission in a non-endemic setting to quantify the potential impact of expanded antibiotic treatment on disease burden and antibiotic use. Through simulations, we evaluated different outbreak scenarios, by varying the reproductive number, care-seeking behavior, and proportion of non-severe cases receiving antibiotics. We found that expanding antibiotic treatment could significantly reduce the final outbreak size under certain outbreak characteristics. In these scenarios, treating non-severely symptomatic infections with antibiotics decreased cholera transmission and, in some cases, the total number of antibiotic doses used. We show that the effectiveness of expanded antibiotic treatment is highly dependent on achieving high care-seeking rates among non-severely symptomatic infections and tailoring the approach to specific outbreak conditions. While expanding antibiotic eligibility could enhance outbreak control in some settings, careful consideration of antibiotic resistance risks is necessary in high-transmission contexts.

## Author summary

Cholera remains a dangerous infectious disease in many parts of the world, with more deaths occurring in more countries in recent years. Most people recover from cholera with simple rehydration. For high-risk and severe cases, antibiotics are used to clear the infection faster. Without antibiotics, people can continue to shed cholera bacteria even after they feel better. Antibiotics stop this bacterial shedding, which decreases how much bacteria is spreading in the community. However, the more antibiotics are used, the greater the likelihood that cholera develops antibiotic resistance, which would make antibiotics no longer an effective treatment. In this study, we use a mathematical model to test two antibiotic prescribing scenarios: a) the current guidelines of antibiotics only for high-risk/severe patients, and b) prescribing antibiotics to more cholera patients, with the idea that this will lead to less spreading of cholera and fewer cases overall. We show treating more types of patients (severely and moderately ill) with antibiotics can lead to smaller outbreaks and fewer doses of antibiotics prescribed under certain outbreak conditions. We also show which parts of the model are most important, and highlight areas that need more scientific research before we can be confident in our results.

## Introduction

Cholera, causxed by the toxigenic bacterium *Vibrio cholerae* O1/O139, remains a significant public health threat [1]. It is characterized by severe acute watery diarrhea that can cause death within hours if left untreated [1,2]. Despite its severe clinical manifestations, the true burden of cholera is not well characterized, as only approximately 10% of cases experience severe symptoms and it has been estimated that the vast majority of infections are asymptomatic or unreported [3–6]. A 2015 global burden analysis estimated 2.86 million cases (uncertainty range 1.3–4 million cases) and 95,000 deaths (uncertainty range 21,000–143,000 deaths) in endemic countries annually [7]. Although the global burden of cholera has been declining in recent decades, a notable resurgence in cases, countries affected, and the case fatality rate has been described since 2021 [8], with 32 countries reporting over 565,000 cases from January to October 2025 [9].

Patterns of cholera transmission vary between endemic regions and non-endemic regions, in part due to protective immunity [10]. Importantly, the estimated durability of natural immunity varies from several months to 10 years [11]. Subclinical cholera infections may confer lower protection than clinical infections [12]. As a result, in endemic regions where local transmission has been detected over the past 3 years, incidence of cholera is highest among young children, as young children are the least likely to have previous exposure and therefore immunity. Whereas in non-endemic (outbreak) settings where cholera does not regularly occur, attack rates among children and adults are similar, as the entire population has a similarly low or non-existent level of pre-existing immunity [13,14].

Cholera is an easily treatable disease, with oral rehydration solution (ORS) serving as the cornerstone of treatment. Non-severe cholera cases can be successfully managed with ORS, which, when administered promptly, can reduce the mortality rate to below 1% [1,6,15,16]. Antibiotics, though available for cholera treatment, are generally reserved for severely dehydrated patients or those with high-risk conditions such as pregnancy or severe acute malnutrition, in part due to concerns about antibiotic resistance [15–17]. While antibiotics reduce the duration of symptoms and stool volume, their use is not typically recommended for non-severely ill infections, as the benefit to the patient is modest relative to the risk of development of antibiotic resistance at the population level [16,18,19]. Antibiotics do however confer benefits related to transmissibility: infections untreated with antibiotics shed *V. cholerae* for up to 10 days after symptom resolution, contributing to community transmission [1]. In contrast, antibiotic treatment reduces the duration of bacterial shedding by up to 90% and likely the concentration of infectious bacteria in the stool, thereby considerably reducing cases’ transmission potential [16,17].

Currently, treatment guidelines reserve antibiotics for cholera patients who are severely dehydrated, having severe challenges in rehydration, or have coexisting conditions (e.g., severe acute malnutrition, pregnancy) [16]. When indicated, the recommended treatment for cholera is a single dose of doxycycline, though azithromycin and ciprofloxacin can be used in certain situations [16]. Antibiotics are often under-regulated in areas where cholera is most likely to occur, and people frequently self-medicate with antibiotics before presenting to formal clinical care [20–22]. These self-medicated antibiotics are often inappropriate to treat cholera and further contribute to the development of antibiotic resistance [20]. Recently, it has been proposed that the reduction in transmissibility associated with antibiotic treatment could offer public health benefits by curtailing outbreak transmission [23]. If expanding antibiotic treatment guidelines to include mild and moderately symptomatic infections reduces transmission, this could lead to fewer infectious and potentially fewer antibiotic doses used over the course of an outbreak.

While modeling methodology continues to advance [24,25], the majority of modern cholera modeling studies continue to use compartmental models, and most include direction and indirect transmission pathways [26]. Many recent models also include separate compartments for hyperinfectious vs lower infectivity individuals [27] When human behavior has been incorporated into cholera math models, it encompasses behavior changes that alter likelihood of disease transmission, such as safer drinking water practices or reducing social contacts [27] While models incorporating antibiotic treatment do exist, they do not separate antibiotic use by disease severity ([28–30], for example). In fact, only 4% of studies included in a recent review of mathematical models of cholera included both symptomatic and asymptomatic cases. [26] Given that cholera antibiotic treatment guidelines are based on symptom severity, simulating possible changes in antibiotic guidelines requires a model that includes symptom severity-specific care-seeking behavior and antibiotic prescribing.

In this study, we develop and apply a compartmental model of cholera transmission to quantify the impact of expanding antibiotic treatment to include mild or moderately symptomatic infections. Our objective was twofold: 1) to assess how treatment of those with moderate/some dehydration could impact the overall burden of cholera in an outbreak as well as the risk of developing antibiotic resistance, and 2) to highlight what additional epidemiological information is most needed to improve our understanding of cholera dynamics, which would improve our ability to evaluate potential treatment policy changes. The main difference in our model compared to previous cholera models is the inclusion of three separate symptom severities, with their own treatment and recovery streams. This was required in order to align with current treatment guidelines and address our primary study question. In the interest of mathematical tractability and to avoid introducing poorly constrained parameters, we do not explicitly model environmental reservoirs. We also only consider medically-prescribed antibiotic treatment, as self-prescribing is unlikely to be affected by medical prescribing policy changes, and is often ineffective for the treatment of cholera anyway. Therefore, we focus on non-endemic outbreak settings.

## Methods

### Model

**Fig 1:**
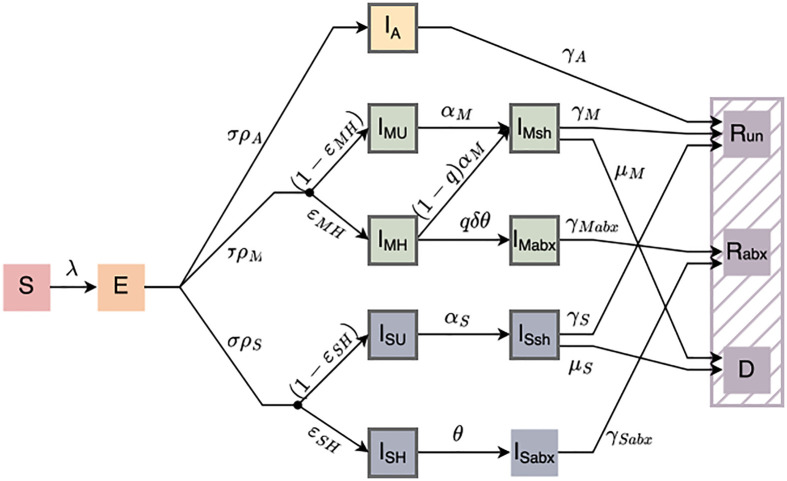
Compartmental diagram of the cholera transmission model. All individuals start as susceptible (S) and become exposed (E) at a rate λ. λ is derived from β, which is in turn derived from the effective reproductive number ($R_{e}$) which is supplied to the model, see Table 1 and Supplement Information. Exposed individuals transition to the infected (I) compartment and we differentiate by symptoms ($I_{A},\;I_{M},\;I_{S}$, asymptomatic, non-severely, or severely symptomatic, respectively) and by care seeking behavior ($I_{U,}I_{H}$, not care seeking and care seeking, respectively). All severely symptomatic infections are treated with antibiotics whereas not all non-severely symptomatic infections who seek treatment receive antibiotics. The proportion of healthcare seeking non-severely symptomatic infections who receive antibiotics is governed by q. Untreated infections, both non-severe and severe, continue to shed for a longer duration following the resolution of symptoms ($I_{Msh},\;I_{Ssh}$), occurring at rate $\alpha_{M},\;\alpha_{S}$, respectively. Non-severely symptomatic infections who are treated with antibiotics continue to shed for a shorter duration following treatment ($I_{Mabx}$), occurring at rate δθ, whereas severely symptomatic infections who are treated with antibiotics remain in a treatment facility and do not contribute to transmission ($I_{Sabx}$). Compartments with a dark grey outline indicate they contribute to transmission. Infectious individuals either recover (R, at rates $\gamma_{A},\gamma_{M},\gamma_{Mabx},\gamma_{S},\gamma_{Sabx}$) or die (D, at rates $\mu_{M},\mu_{S}$) and we differentiate between individuals who recover without antibiotic treatment ($R_{un}$) and those who recover with antibiotics ($R_{abx}$) to compare the number of doses used under different treatment scenarios.

To assess the impact of expanded antibiotic treatment guidelines on cholera outbreaks, we developed and analyzed a compartmental model of cholera transmission in a non-endemic setting (**Fig 1**). Susceptible (S) individuals, with no immunity from prior infection or vaccination become exposed (E) upon successful transmission and then progress to one of the infectious compartments (I). We differentiate within the infectious compartments based on symptomatology: asymptomatic ($I_{A}$), non-severely symptomatic infections, including both mild and moderately symptomatic ($I_{M}$), (hereafter referred to collectively as “moderate”), and severely symptomatic ($I_{S}$) as defined by dehydration. Additionally, we differentiate within the symptomatic infections based on care seeking behavior: seeking care ($I_{H}$) or not seeking care ($I_{U}$).

**Table 1:** Summary of the model parameters (and available sources) used in model.

Parameter	Meaning	Value/Range	Source
$R_{e}$	Effective reproductive number	Low: 1.3 – 1.5 Intermediate: 1.5–2.0 High: 2.3 – 2.8	[2,31–39]
1/σ	Latent period	1.3-1.6 days	[40]
${\rho_{A},\;\rho }_{M}{,\rho }_{S}$	Proportion of infected individuals in each symptom class such that: ${\rho_{A}+\;\rho }_{M}+\rho_{S}=\text{1}$	Asymptomatic: 0.65–0.85 (*see supplement for additional ranges*) Non-severe: 0.09 – 0.28 Severe: 0.05 –0.1	[41–44]
$\varepsilon_{MH}{,\;\varepsilon }_{SH}$	Proportion of non-severe or severely symptomatic infected individuals who seek care, respectively (proportion care-seeking)	Non-severe: 0–1, varies based on scenario Severe: 0.5 –0.9	[44,45]
$\alpha_{M},\;\alpha_{S}$	Time to developing symptoms for untreated infections (in days)	Non-severe: 2.5-5 days Severe: 3.34-10 days	[46,47]
1/θ	Time to treatment for severe infections (in days)	0.25-1.5 days	Varies based on patient behavior and available resources
1/δ	Relative increase in time to treatment for non-severe relative to severe infections	2	Based on patient behavior and available resources
q	Non-severely symptomatic infection treatment effort (*see supplement for additional details*)	Varies by scenario	
$\text{1}/\gamma_{A},\text{1}/\gamma_{M},\text{1}/\gamma_{S}$	Time to recovery without antibiotic treatment (in days)	Asymptomatic: 3.4-10 days Non-severe: 4.2-10 days Severe: 5–10 days	[45–50]
$\text{1}/\gamma_{M_{abx}},\text{1}/\gamma_{S_{abx}}$	Time to recovery with antibiotic treatment (in days)	Non-severe: 1.67-3.34 days Severe: 1 day	[45–50]
$\text{1}/\mu_{M},\text{1}/\mu_{S}$	Time to death (in days)	Asymptomatic: do not die Non-severe: 1–2 days Severe: 0.5 –1 day	[46–48,45]

We do not attempt to parameterize care seeking behavior, rather, we vary the proportion of symptomatic infections who seek care and the rate at which symptomatic care-seekers receive antibiotic treatment between simulations. Due to the nature of their symptoms, we assume that all severely symptomatic infections who seek care ($I_{SH}$) receive antibiotic treatment ($I_{Sabx}$) and that severely symptomatic infections are much more likely to seek treatment. We model the effect of varying the proportion of non-severely symptomatic infections that seek ($I_{MH}$) and receive antibiotic treatment ($I_{Mabx}$). We assume that asymptomatic infections never seek treatment and thus do not further divide this compartment by treatment. Further, we do not model treatment adherence, since antibiotic treatment for cholera is a single dose regimen. We assume this single dose is given by a trained health professional at the place of care-seeking and is appropriate for the age, weight, etc. of the patient, at the minimum effective quantity (*i.e.,* mg/kg) [16]. We continue the assumption used in the current cholera treatment guidelines, namely that fewer doses (equivalent to fewer people treated with antibiotics) results in less development of resistance.

We assume that all infected individuals (including asymptomatic infections) contribute to transmission until they are either treated with antibiotics or their infection resolves. Since infected individuals untreated with antibiotics continue to shed *V. cholerae* for up to 10 days post-symptom resolution [51], we assume that they continue to contribute to transmission after the conclusion of symptoms, but at a reduced rate ($I_{Msh},\;I_{Ssh}$, for non-severely and severely symptomatic infections, respectively). For non-severely symptomatic infections treated with antibiotics ($I_{Mabx}$), we assume that they contribute to transmission after receiving antibiotic treatment but at a reduced rate and for a reduced duration. In contrast, we assume that severely symptomatic infections that receive care ($I_{Sabx}$) are admitted to a treatment center and therefore no longer shed into the community, and thus do not contribute to transmission after receiving antibiotic treatment. In general, we parameterize our model to ensure key rates for non-severely symptomatic infections, such as treatment seeking (θ) and proportion in each symptom class ($\rho_{A},\;\rho_{M},\;\rho_{S}$) are proportional to those for severe infections. For full model equations, *see* S1 File. See [23] for a detailed derivation of the reproductive number for this model.

Finally, we assume that cholera transmission is adequately captured by human-to-human transmission. As Wang et. al. [27] discuss while citing [52,53], hyper-infectivity in environmental-to-human transmission may not be meaningfully different than human-to-human transmission, especially when interested in transmission shortly after the onset of symptoms. Because our goal is to evaluate a public health intervention that acts on a short time-scale relative to symptom onset, and for mathematical tractability, we do not include compartments for an environmental reservoir such as contaminated water supply. Consistent with this assumption, we only consider non-endemic outbreaks, with infection rapidly spreading in a naïve population, and assume births, deaths, disease seasonality, and the development of antibiotic resistance etc. are negligible over the modeled time period.

### Scenarios

We evaluate the impact of treating mild and moderately (non-severe) symptomatic infections with antibiotics over different outbreak scenarios. These scenarios are characterized by varying the effective reproductive number, the proportion care seeking, and proportion treated with appropriate antibiotics.

Because cholera outbreaks have high variability in the reported effective reproductive number by outbreak setting, we designed three scenarios based on these different transmission characteristics at the start of the outbreak: low $R_{e}=\text{1}.\text{3}-\text{1}.\text{5}$ and intermediate $R_{e}=\text{1}.\text{6}-\text{2}.\text{0}\;$ based off outbreaks from Africa [33–37] and high $R_{e}=\text{2}.\text{3}-\text{2}.\text{8}$ based off outbreaks from the Americas [31,32,38,39].

Our primary interest lies in understanding the impact of expanding antibiotic treatment eligibility guidelines to include non-severely symptomatic infections. However, the proportion of non-severely symptomatic infections that receive appropriate antibiotics is governed both by the proportion that seek care ($\varepsilon_{MH}$) as well as the proportion of care-seeking non-severely symptomatic infections who are treated (q). Care-seeking behavior among non-severely symptomatic infections is not well characterized and is likely influenced by the current treatment guidelines that limit antibiotics to severely symptomatic infections. As such, we explore five scenarios for care seeking: 5%, 25%, 50%, 75%, and 100% of non-severely symptomatic infections seeking care (five values of $\varepsilon_{MH})$.

Within the scenarios described above, we also vary the proportion of non-severely symptomatic infections who have sought care that receive antibiotic treatment (“proportion treated with antibiotics,” derived from q, see supplement) from no one (current treatment guidelines) to everyone (all care-seeking non-severely symptomatic infections receive antibiotics).

### Simulations

We simulate our model using R statistical software [54] over the range of parameter values presented in Table 1. Since we are modeling a non-endemic outbreak, we initialize our model with 0.01% of the entirely susceptible population infected. We simulate each scenario 1000 times until stochastic extinction.

Many key parameters are not well characterized for cholera, as evidenced by the wide range of many parameters presented in Table 1. As such, we use Latin hypercube sampling (LHS) to sample across the full parameter uncertainty range [55,56]. LHS ensures comprehensive exploration of the parameter space by sampling each interval only once, allowing for the distinction between variability in outcomes driven by the stochastic nature of the model from that arising due to parameter uncertainty [56]. In other words, if our range of parameter value encompasses the true parameter value, the outcome metric should also encompass the truth. We constrain all parameters to ensure that no draws from the LHS yield epidemiologically implausible values.

### Epidemiologic outcomes

We evaluate the scenarios across several metrics, including the total number of infections (final size), the total number of antibiotic doses used over the course of the outbreak, the number of infections under expanded treatment minus the number of infections under current treatment guidelines (infections averted), as well as the number of doses used under expanded treatment minus the number of doses used under current treatment guidelines (additional doses used).

## Results

We created a compartmental model of cholera transmission in a non-endemic setting to assess the population level effects of expanded antibiotic treatment guidelines to include moderately symptomatic cases.

Through simulation, we show that expanding antibiotic treatment guidelines to include non-severely symptomatic infections can substantially reduce the burden of cholera in low and intermediate transmission settings, especially when rates of care-seeking behavior is high. In high transmission settings, the impact of expanded antibiotic treatment guidelines is less pronounced but still offers marginal reductions in the final outbreak size.

**Fig 2:**
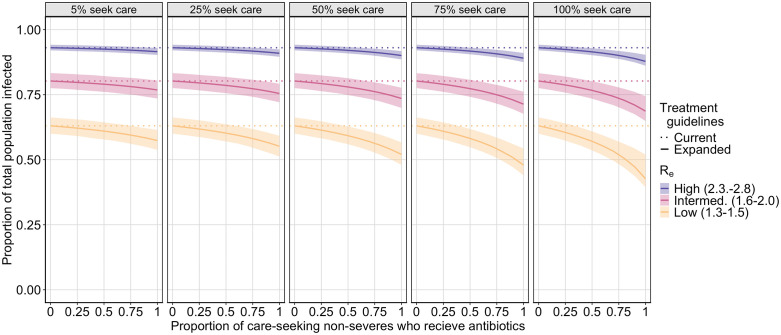
Plot of the final outbreak size by the proportion of care-seeking non-severely symptomatic infections treated with antibiotics. Each plot shows the final proportion of the population infected by the proportion of care-seeking non-severely symptomatic infections who receive treatment for low (yellow), intermediate (pink), and high (purple) effective reproductive numbers, for a different percent of non-severely symptomatic infections who seek treatment (5%, 25%, 50%, 75%, 100%). The solid line indicates the mean estimate under expanded treatment guidelines, the shaded region represents the 25% and 75% quantiles, and the dashed line shows the final size of the outbreak under current antibiotic treatment guidelines (treating no non-severely symptomatic infections).

We find that treating non-severely symptomatic infections with antibiotics decreases the final size of the outbreak across all care seeking scenarios and transmission scenarios except in high $R_{e}$ outbreaks with very low proportion of care seeking (5%) among non-severely symptomatic infections (**Fig 2**). While treating non-severely symptomatic infections with antibiotics almost always decreases the final outbreak size, the effect is largest when more non-severely symptomatic infections seek care and receive appropriate antibiotics as well as when the effective reproductive number is low.

While treating non-severely symptomatic infections with antibiotics can substantially reduce cholera transmission, we show that expanded antibiotic eligibility alone is not sufficient to significantly reduce transmission unless it is coupled with high care-seeking rates.

**Fig 3:**
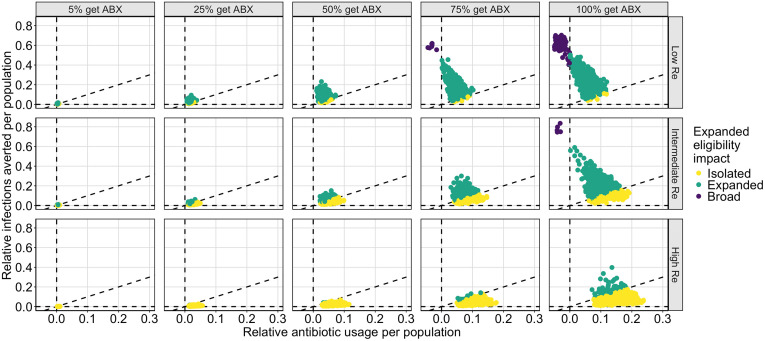
Plot of the population-level impact of expanded antibiotic treatment guidelines. Each plot compares the relative infections averted per population to the relative antibiotic usage per population. The relative infections averted is calculated as the ratio of the number of infections in a given scenario (combination of R_e_ and antibiotic deployment in moderates), divided by the number of infections under the current antibiotic treatment guidelines (only severely-symptomatic infections receive antibiotics), using the same LHS sampled parameters and normalized by population size. Similarly, the relative antibiotic usage is calculated as the ratio of antibiotic doses used in a given scenario, divided by the number of antibiotic doses used under current antibiotic treatment guidelines (only severely-symptomatic infections receive antibiotics), again using the same LHS sampled parameters and normalized by population size. Each plot represents a different proportion of non-severely symptomatic infections seeking care who receive antibiotic (ABX) treatment (5%, 25%, 50%, 75%, 100%) and a different $R_{e}$ scenario (low ($R_{e}=\text{1}.\text{3}-\text{1}.\text{5}$), intermediate ${(R}_{e}=\text{1}.\text{6}-\text{2}.\text{0}$), high ($R_{e}=\text{2}.\text{3}-\text{2}.\text{8}$)). The outcomes are split into three regions by the impact of expanded eligibility criteria: between the dashed line along the x-axis (expanding criteria averts no infections) and the diagonal dashed line (each addition dose of antibiotics deployed averts one infection), yellow points represent simulations in which expanded eligibility only has isolated benefits; between the diagonal dashed line and the vertical dashed line (no additional doses are used to avert infections), green points represent simulations in which expanded eligibility results in each dose preventing more than one additional infection; and in the region left of the vertical dashed line, purple points represent simulations in which expanded eligibility results in fewer doses used over the course of the outbreak than compared to current antibiotic treatment guidelines.

The development of antibiotic resistance remains a significant concern that informs current cholera treatment guidelines. Although we did not explicitly model the evolution of resistance, we use the number of antibiotic doses administered as a proxy for selective pressure. As such, we evaluated our scenarios based on the number of antibiotic doses used and categorized them by the scale of impact: isolated, expanding, and broad. Our simulations reveal three distinct regions within the range of tested parameters (**Fig 3**). In one region, extending antibiotic treatment guidelines to include non-severely symptomatic infections confer only isolated benefits, as less than one infection is averted per additional antibiotic dose over the course of the outbreak (yellow points, **Fig 3**). The next region is such that extending antibiotic treatment criteria provides an expanding benefit, whereby treating non-severely symptomatic infections with antibiotics reduces transmission sufficiently such that each additional dose averts more than one infection (green points, **Fig 3**). In the final region extending antibiotic treatment criteria results in broad benefits where fewer antibiotic doses are used over the course of an outbreak compared to current treatment practices (purple points, **Fig 3**). Which region a simulation falls into is governed by both $R_{e}$ and the proportion of non-severely symptomatic infections treated with antibiotics. As $R_{e}$ increases, the benefits of treating non-severely symptomatic infections with antibiotics shifts from population-level benefits and antibiotic dose reduction to primarily individual-level benefits. Likewise, as the proportion of non-severely symptomatic infections treated with antibiotics increases, the population-level benefit of expanded antibiotic use becomes more pronounced.

**Fig 4:**
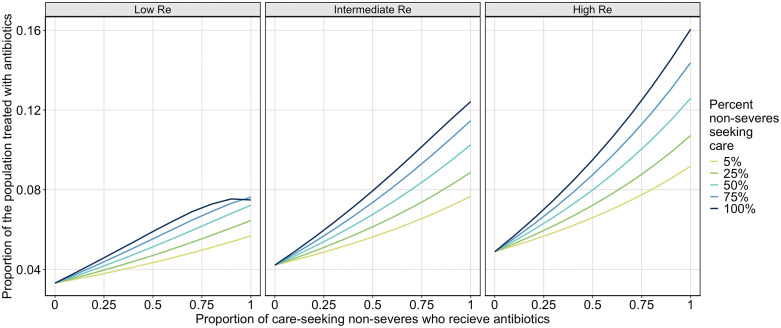
Plot of population-level antibiotic treatment. Each plot shows the proportion of the total population who receive appropriate antibiotics, by the proportion of care-seeking non-severely symptomatic infections that receive appropriate antibiotics, separated by $R_{e}$ scenario (low ($R_{e}=\text{1}.\text{3}-\text{1}.\text{5}$), intermediate ${(R}_{e}=\text{1}.\text{6}-\text{2}.\text{0}$), high ($R_{e}=\text{2}.\text{3}-\text{2}.\text{8}$)). For each $R_{e}$ scenario, we show five values for the proportion of non-severely symptomatic infections that seek care, 5% (yellow), 25% (green), 50% (teal), 75% (blue), 100% (navy), and vary the proportion of care-seeking non-severely symptomatic infections who receive antibiotics from 0 – 100%.

To further explore the impact of expanded treatment on the number of antibiotic doses used over the course of the outbreak, we evaluate how the proportion of care-seeking non-severe individuals who receive appropriate antibiotics impacts the proportion of the total population who receive appropriate antibiotics (**Fig 4**). Recall that antimicrobial treatment for cholera involves a single dose of antibiotics per patient per illness. As with infections averted, when the transmission rate is lower and when more non-severely symptomatic infections are treated, treating non-severely symptomatic infections with antibiotics has greater public health benefits. Indeed, when $R_{e}$ is low, increasing the proportion of non-severely symptomatic infections treated with antibiotics results in fewer antibiotic doses used. For intermediate and high transmission settings, increasing the proportion of non-severely symptomatic infections treated with antibiotics only results in an increase in the proportion of the total population treated with antibiotics.

## Discussion

This study builds on prior research suggesting that, under certain circumstances, expanding antibiotic treatment of cholera cases can provide population-level benefits by reducing transmission and limiting an outbreak size such that fewer antibiotic doses are used over the course of the outbreak. We also highlight how a better understanding of the epidemiology of cholera, especially reproductive number and care-seeking behavior, are critical for accurate treatment policy evaluation. While previous models have included antibiotic treatment, [29,30] they have not separated infections by symptom severity. Other models also explicitly explore the dynamics of antibiotic resistance in cholera outbreaks, [29,30] but do not separate medically-guided antibiotic use by symptom severity. In our model, we have separated infections into symptom-severity-specific streams, with unique care-seeking and antibiotic treatment. We also only considered medically-guided antibiotic use, and did not incorporate self-prescribing of appropriate or inappropriate antibiotics. While this introduces some bias into our model, it allows us to directly simulate different antibiotic treatment policies. We previously proposed a theoretical mechanism for the relationship between antibiotic treatment policy and disease burden [23]. In this paper we identified the specific conditions where these benefits can be achieved for cholera. Our findings show how treating non-severe cholera cases with antibiotics can help control outbreaks and highlight the critical role of linking these cases to healthcare in achieving impact. We detail how outbreak specific quantitates such as the effective reproductive number and the proportion of non-severely symptomatic infections that seek care impact the effectiveness of this strategy. Finally, we present a broad range of estimated effects consistent with the parameters currently available in the literature, and detail how these effects could become clearer with more refined knowledge of cholera epidemiology.

The effectiveness of expanding antibiotic treatment criteria improves as the proportion of non-severely symptomatic infections seeking care increases. In the majority of outbreaks which experience low to intermediate transmission rates (measured by the reproductive number), such as those seen in most cholera outbreaks [21,22,24,25,57], reducing transmission can substantially lower the proportion of the population infected, supporting the theoretical findings that expanded antibiotic access can enhance outbreak control.

A key distinction emerges between low and high $R_{e}$ settings. In lower $R_{e}$ settings, treating a high proportion of non-severely symptomatic infections reduces both cholera burden and number of antibiotic doses used over the course of the outbreak. However, as $R_{e}$ increases, these benefits disappear, though an intermediate range exists where each additional dose of antibiotics used averts more than one infection, still providing a public health benefit. In high $R_{e}$ settings, treating non-severely symptomatic infections may reduce the outbreak size, but it requires treating a larger portion of the population. These results suggest that treating non-severe cholera cases may be effective in low $R_{e}$ settings but may carry a risk of antibiotic resistance in high-transmission contexts, underscoring the importance of tailoring treatment strategies to the specific outbreak context. In this paper, the only intervention we consider is expanding antibiotic treatment guidelines, however this could be combined with other interventions as a component in a large outbreak containment strategy.

Across all simulations, the most important factor governing the effectiveness of treating non-severely symptomatic infections with antibiotics is achieving high antibiotic treatment rates. Our related analysis explicitly demonstrates the importance of reproductive number and proportion of moderate cases receiving antibiotics in driving the effectiveness of expanded antibiotic eligibility. [23] While we do not directly parameterize care-seeking or treatment availability, we do incorporate variation in antibiotic treatment rates by varying the proportion of symptomatic infections who seek care and the rate at which they receive antibiotics. Often the focus on clinical care during outbreaks is at cholera treatment centers, typically located at secondary and tertiary health structures. While there are gaps in our understanding of care seeking behavior for non-severely symptomatic infections, experience in endemic settings suggest that most people with non-severe disease will seek care at pharmacies or lower-level health facilities, frequently obtaining inappropriate or non-indicated antibiotics [21,22,20,58,59]. Developing strategies to expand access to appropriate antibiotics at decentralized oral treatment posts or pharmacies could have a critical impact on achieving sufficiently high coverage of antibiotics among non-severely symptomatic infections to reduce cholera transmission while also improving antibiotic stewardship. Expanded access could be coupled with the use of rapid diagnostic tests for cholera to improve the specificity of targeting people with diarrhea caused by *Vibrio cholerae* O1. Addressing barriers to care seeking behavior either through outreach or through expanded access to appropriate antibiotics through pharmacies is necessary to achieve sufficiently high treatment rates. Cholera contact targeted interventions are an active and promising area of research, and may offer a strategy to achieve these desired antibiotic treatment rates [60–62].

An important limitation of our study is the fact that many of the key parameters necessary for modeling cholera have not been well characterized in the literature. Accurate estimates of, in particular, $R_{e}$ and the proportion of non-severely symptomatic infections seeking treatment are crucial and would require purposefully designed epidemiological studies in a well-characterized population with comprehensive diagnostic testing in order to refine the parameters used in our model. We only consider non-endemic settings, assuming no prior immunity. Further analysis is needed to assess how prior immunity or vaccination affects our results, but these analyses will require additional parameters that are also not well characterized in the literature. While we do not model immunity directly, in a supplementary analysis, we evaluate the impact of varying the ratio of asymptomatic to symptomatic infections, one of the expected main differences between an outbreak setting to an endemic setting. We find that our final size results are robust to variations in the proportion asymptomatic, but for outbreaks with a very high proportion of infections that are asymptomatic (consistent with highly endemic settings), the broad impacts from expanding antibiotic eligibility disappear (**Fig A in** **S1 File**). Conversely, for outbreaks with a very low proportion of infections that are asymptomatic (consistent with totally naïve settings), not only are broad and expanded impacts more pronounced (**Fig B in** **S1 File**), in low $R_{e}$ outbreak settings, expanding antibiotic treatment guidelines has such a substantial benefit that it halts transmission so effectively that it can prevent an outbreak from ever taking off (**Fig C in** **S1 File**). Thus, we expect expanded treatment guidelines may have a smaller impact in endemic settings than outbreak settings.

Additionally, we only consider three symptom categories – severely symptomatic, non-severely symptomatic, and asymptomatic infections. A more granular distinction within the non-severely symptomatic compartment (e.g., mild and moderate) may refine treatment estimates, but again the literature lacks this level of specificity on necessary parameters. Since infectiousness correlates with symptom severity [5], it may suffice to target the most symptomatic non-severely symptomatic infections. Achieving a high enough treatment coverage may be a barrier to utilizing this strategy as an outbreak containment strategy, particularly in resource-limited settings where both antibiotics and healthcare infrastructure may be constrained.

Future model expansions should explore the impact of adding environmental reservoirs to the model (hyper-infective and lower infectivity bacterial contamination, as well as a reservoir for re-introduction) to better account for environmental-to-human transmission. This would have the added benefit of allowing a side-by-side comparison of expanded antibiotic eligibility and water, sanitation, & hygiene interventions on outbreak dynamics. Addition model expansions should also include more nuanced antibiotic prescribing, including self-prescribing, administration of antibiotics against which cholera is not sensitive, and feedback loops for the development of antibiotic resistance. Finally, future work on infectious disease model development will need to explore the feasibility and utility of incorporating artificial intelligence and similar technologies into infectious disease modeling studies [63].

Our study builds on phenomenological findings which suggest that treating non-severely symptomatic infections can reduce the number of antibiotic doses needed and may therefore lower the selective pressure for cholera to develop resistance. However, uncertainties in parameter estimates raise concerns about balancing these benefits with the risks of promoting antimicrobial resistance, which would hinder future cholera treatment and outbreak response efforts. Future studies aimed at improving characterization of key parameters or a clinical trial aimed at validating these model results and assessing the feasibility of implementing expanded treatment guidelines are a critical next step towards implementing these findings. Careful monitoring of key parameters and resistance patterns will be crucial in ensuring that the benefits of expanded antibiotic treatment criteria do not increase the risk of developing antibiotic resistant cholera.

## Supporting information

S1 File Material. includes additional details on model formulation, including the differential equations, mathematical expressions for the force of infection and proportion of care-seeking non-severely symptomatic infections that receive antibiotics, and the symptom-treatment specific transmission parameters. Additional results from sensitivity analysis are also included. [57,46–48,64,65]. Table A: Summary of the transmission parameters (and available sources) used in model. Fig A: Plot of the population-level impact of expanded antibiotic treatment guidelines with 90%-99% asymptomatic infections. Fig B: Plot of the population-level impact of expanded antibiotic treatment guidelines with 17%-33% asymptomatic infections. Fig C: Plot of the final outbreak size by the proportion of care-seeking non-severely symptomatic infections treated with appropriate antibiotics for small proportion asymptomatic (17%-33%).
(PDF)
