# Supplementary material for: Quantifying the population-level impact of expanded antibiotic treatment for cholera outbreak management
Source: PLoS Comput Biol. 2026 Feb 18;22(2):e1013980. doi: 10.1371/journal.pcbi.1013980 (PMC12931884; doi:10.1371/journal.pcbi.1013980)
Supplement: S1 File — Additional results from sensitivity analysis are also included. [57,46–48,64,65]. Table A: Summary of the transmission parameters (and available sources) used in model. Fig A: Plot of the population-level impact of expanded antibiotic treatment guidelines with 90%-99% asymptomatic infections. Fig B: Plot of the population-level impact of expanded antibiotic treatment guidelines with 17%-33% asymptomatic infections. Fig C: Plot of the final outbreak size by the proportion of care-seeking non-severely symptomatic infections treated with appropriate antibiotics for small proportion asymptomatic (17%-33%). (PDF) [file pcbi.1013980.s001.pdf]

Title: Quantifying the population-level impact of expanded antibiotic treatment for cholera outbreak management

Authors: Sharia M. Ahmed, PhD, MPH[$*,1,2], Cormac R. LaPrete, MS[$,3], Iza Ciglenecki, PhD[4], Andrew Azman, PhD[5], Daniel T. Leung, MD[6], Lindsay T. Keegan, PhD[1]

[$] *Indicates these authors contributed equally*
[*] *Indicates the corresponding author: Sharia.m.ahmed@emory.edu*

Corresponding author contact information:
Sharia M Ahmed
Sharia.m.ahmed@emory.edu
404-727-1694
Emory Rollins School of Public Health
1518 Clifton Rd NE
Atlanta, GA 30322
USA

Affiliations:
[1] Division of Epidemiology, University of Utah School of Medicine, Salt Lake City, Utah, United States of America
[2] Department of Epidemiology, Emory University, Atlanta, Georgia, United States of America
[3] Department of Mathematics, University of Utah College of Science , Salt Lake City, Utah, United States of America
[4] Médecins sans Frontières, Geneva, Switzerland
[5] Department of Epidemiology, Johns Hopkins Bloomberg School of Public Health, Baltimore, Maryland, United Stated of America
[6] Division of Infectious Diseases, University of Utah School of Medicine, Salt Lake City, Utah, United States of America

Supplemental Information

Methods

Model Equations

$$\frac{dE}{dt} = \lambda S - \sigma E$$

$$\frac{dI_A}{dt} = \sigma p_A E - \gamma_A I_A$$

$$\frac{dI_{MU}}{dt} = \sigma p_M (1 - \varepsilon_{MH}) E - \alpha_M I_{MU}$$

$$\frac{dI_{MH}}{dt} = \sigma p_M \varepsilon_{MH} E - \left((1-q)\alpha_M + q\delta\theta\right) I_{MH}$$

$$\frac{dI_{M_{sh}}}{dt} = \alpha_M I_{MU} + (1-q)\alpha_M I_{MH} - (\gamma_M + \mu_M) I_{M_{sh}}$$

$$\frac{dI_{M_{abx}}}{dt} = q\delta\theta\, I_{MH} - \gamma_{M_{abx}} I_{M_{abx}}$$

$$\frac{dI_{SU}}{dt} = \sigma p_S (1 - \varepsilon_{MS}) E - \alpha_S I_{SU}$$

$$\frac{dI_{SH}}{dt} = \sigma p_S \varepsilon_{SH} E - \theta I_{SH}$$

$$\frac{dI_{S_{sh}}}{dt} = \alpha_S I_{SU} - (\gamma_S + \mu_S) I_{S_{sh}}$$

$$\frac{dI_{S_{abx}}}{dt} = \theta\, I_{SH} - \gamma_{S_{abx}} I_{S_{abx}}$$

$$\frac{dR_{abx}}{dt} = \gamma_{M_{abx}} I_{M_{abx}} + \gamma_{S_{abx}} I_{S_{abx}}$$

$$\frac{dR_{un}}{dt} = \gamma_A I_A + \gamma_M I_{M_{sh}} + \gamma_S I_{S_{sh}}$$

$$\frac{dD}{dt} = \mu_M I_{M_{sh}} + \mu_S I_{S_{sh}}$$

Force of infection, lambda

As derived from Laprete et al., the force of infection is given by the following mathematical expression:

$$\lambda = \beta\left(I_{S_{SyU}} + I_{S_{SyT}}\right) + \nu_{sh}\beta I_{S_{Sh}} + \nu_A \beta I_{A_{Sh}} + \nu_M \beta\left(I_{M_{SyU}} + I_{M_{SyT}}\right) + \nu_{sh}\nu_M \beta I_{M_{Sh}} + \nu_M \nu_{abx} \beta I_{M_{abx}}$$

Proportion of care-seeking non-severely symptomatic infections that receive antibiotics

As derived from LaPrete et al., the proportion of care-seeking non-severely symptomatic infections that receive antibiotics, M_{abx}, is given by:

$$M_{abx} = \frac{q\delta\theta}{(1-q)\alpha_M + q\delta\theta}$$

Table A in S1 Material: Summary of the transmission parameters (and available sources) used in model.

Parameter	Meaning	Value	Source
ν_A	Reduction in infectiousness, relative to untreated severe infections, for being asymptomatic	0.15–0.35	Expert elicitation
ν_M	Reduction in infectiousness, relative to untreated severe infections, for being non-severely symptomatic	0.45–0.75	Expert elicitation
ν_{sh}	Reduction in infectiousness, relative to untreated severe infections, for no longer being symptomatic but still shedding	0.3–0.6	[39–41]
ν_{abx}	Reduction in infectiousness, relative to untreated severe infections, for having received antibiotics but still shedding	0.3–0.7	Expert elicitation

For key parameters that were unavailable from the current scientific literature, we polled World Health Organization (WHO) Global Task Force on Cholera Control (GTFCC) members over the multiple years of model development. We presented multiple interim model products to WHO GTFCC members to obtain feedback or suggestions for changes in model parametrization.

Assessment of the ratio of asymptomatic to symptomatic infections: Although our study aims to explore non-endemic settings, since the ratio of asymptomatic to symptomatic infections varies by location, we conducted an additional sensitivity analysis to quantify the impact of the ratio of asymptomatic to symptomatic infections on disease burden and number of antibiotic doses used under expanded antibiotic treatment guidelines. Our results in the main text show asymptomatic infections representing 65%-85% of all infections. Here we explore increasing this ratio to 90%-99%, as was reported in Bangladesh[56] and lowering it to 17%-33%, as was found in Haiti [57].

Results

In a supplementary analysis, we vary the ratio of asymptomatic to symptomatic infections from 65%-85% up to 90%-99%, as well as down to 17%-33%. Overall, we find that our final size results are robust to changes in the ratio of asymptomatic to symptomatic infections and that treating non-severely symptomatic infections reduces the final size regardless of this ratio. However, we find that the broad impacts from expanded eligibility disappear for very high proportion asymptomatic (90%-99%) (**Figure A in S1 Material**). Simultaneously, for very high proportion of asymptomatic, we find that the total proportion of the population receiving antibiotics is very small, since most infections are asymptomatic and

therefore do not seek treatment. Conversely, when asymptomatic infections represent a smaller proportion of the population (17%-33%), the proportion of simulations with expanded or broad impacts from expanded eligibility become more pronounced (**Figure B in S1 Material**). Indeed, for some simulations, the benefit is so substantial that it can halt the outbreak before it can take off (**Figure C in S1 Material**).

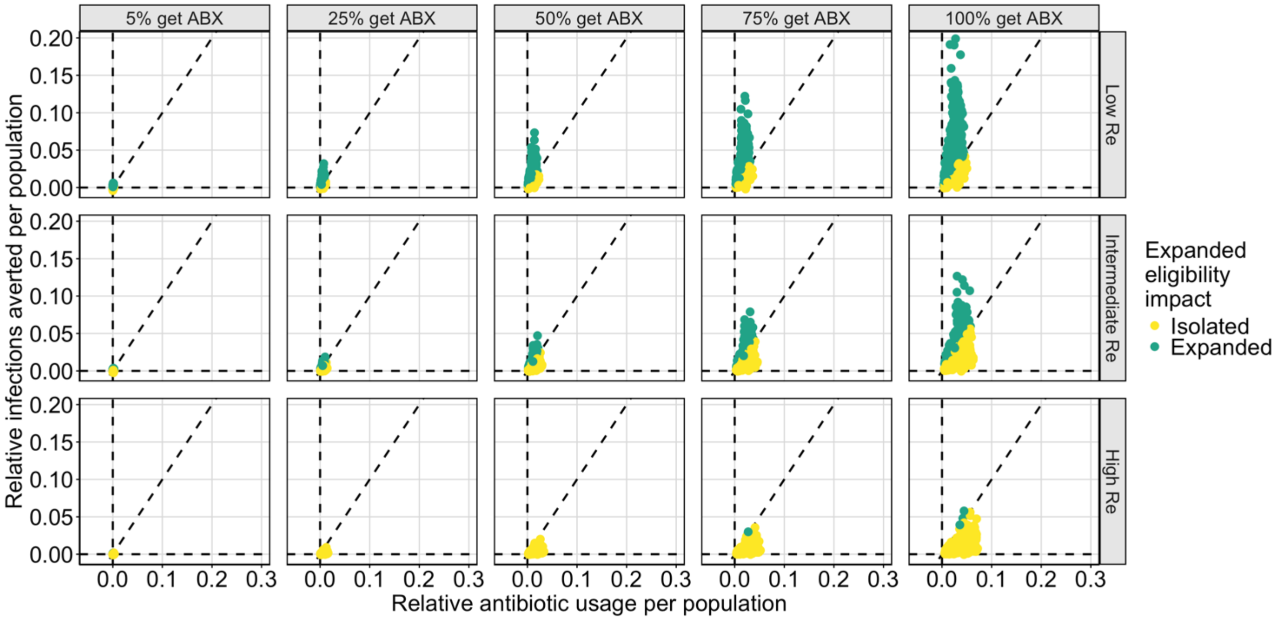

Figure A in S1 Material: Plot of the population-level impact of expanded antibiotic treatment guidelines with 90%-99% asymptomatic infections. Each plot compares the relative infections averted per population to the relative antibiotic usage per population. The relative reduction in infections is calculated by the ratio of the number of infections in each expanded antibiotic treatment scenario presented compared to the simulation of current antibiotic treatment guidelines (treating no non-severely symptomatic infections with antibiotics) using the same LHS sampled parameters, normalized by population size. Similarly, the relative antibiotic usage is calculated by the ratio of antibiotic doses used in each scenario presented compared to the simulation of current antibiotic treatment guidelines (treating no non-severely symptomatic infections with antibiotics) using the same LHS sampled parameters, normalized by population size. Each plot represents a different proportion of non-severely symptomatic infections seeking care who receive antibiotic treatment (5%, 25%, 50%, 75%, 100%) and a different R_e scenario (low ($R_e = 1.3 - 1.5$), intermediate ($R_e = 1.6 - 2.0$), high ($R_e = 2.3 - 2.8$)). The outcomes are split into three regions by the impact of expanded eligibility criteria: between the dashed line along the x-axis (expanding criteria averts no infections) and the diagonal dashed line (each addition dose of antibiotics deployed averts one infection), yellow points represent simulations in which expanded eligibility only has isolated benefits; between the diagonal dashed line and the vertical dashed line (no additional doses are used to avert infections), green points represent simulations in which expanded eligibility results in each dose preventing more than one additional infection; and in the region left of the vertical dashed line, purple points represent simulations in which expanded eligibility results in fewer

doses used over the course of the outbreak than compared to current antibiotic treatment guidelines.

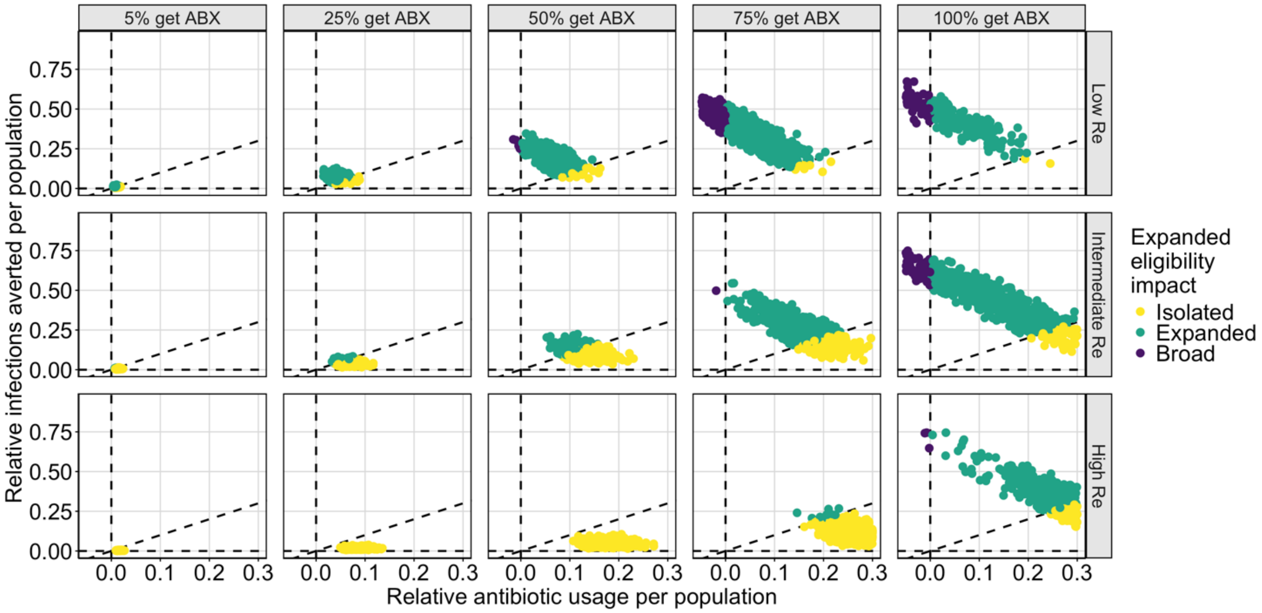

Figure B in S1 Material: Plot of the population-level impact of expanded antibiotic treatment guidelines with 17%-33% asymptomatic infections. Each plot compares the relative infections averted per population to the relative antibiotic usage per population. The relative reduction in infections is calculated by the ratio of the number of infections in each expanded antibiotic treatment scenario presented compared to the simulation of current antibiotic treatment guidelines (treating no non-severely symptomatic infections with antibiotics) using the same LHS sampled parameters, normalized by population size. Similarly, the relative antibiotic usage is calculated by the ratio of antibiotic doses used in each scenario presented compared to the simulation of current antibiotic treatment guidelines (treating no non-severely symptomatic infections with antibiotics) using the same LHS sampled parameters, normalized by population size. Each plot represents a different proportion of non-severely symptomatic infections seeking care who receive antibiotic treatment (5%, 25%, 50%, 75%, 100%) and a different R_e scenario (low ($R_e = 1.3 - 1.5$), intermediate ($R_e = 1.6 - 2.0$), high ($R_e = 2.3 - 2.8$)). The outcomes are split into three regions by the impact of expanded eligibility criteria: between the dashed line along the x-axis (expanding criteria averts no infections) and the diagonal dashed line (each addition dose of antibiotics deployed averts one infection), yellow points represent simulations in which expanded eligibility only has isolated benefits; between the diagonal dashed line and the vertical dashed line (no additional doses are used to avert infections), green points represent simulations in which expanded eligibility results in each dose preventing more than one additional infection; and in the region left of the vertical dashed line, purple points represent simulations in which expanded eligibility results in fewer doses used over the course of the outbreak than compared to current antibiotic treatment guidelines.

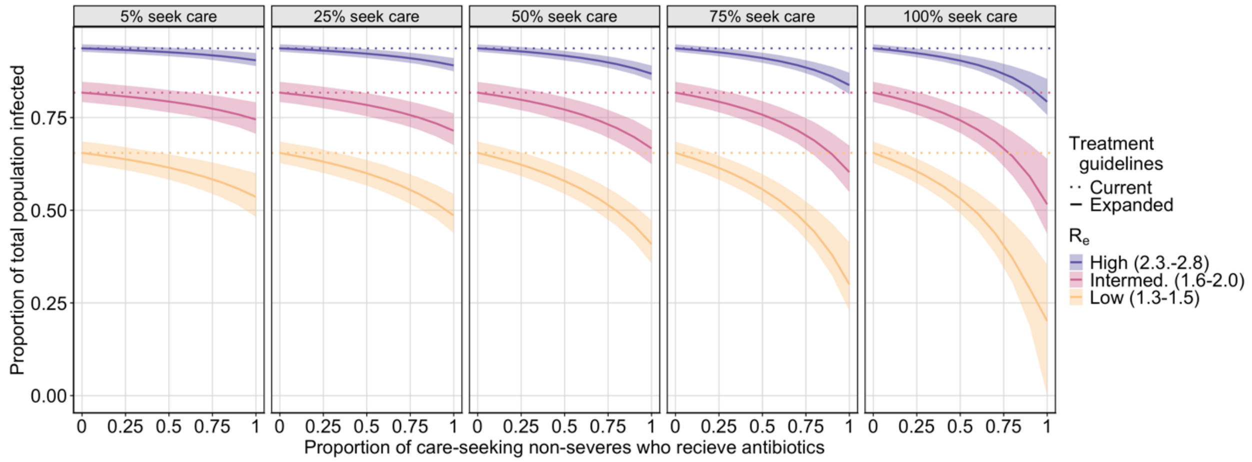

Figure C in S1 Material: Plot of the final outbreak size by the proportion of care-seeking non-severely symptomatic infections treated with appropriate antibiotics for small proportion asymptomatic (17%-33%). Each plot shows the final proportion of the population infected by the proportion of care-seeking non-severely symptomatic infections who receive appropriate antibiotic treatment for low (yellow), intermediate (pink), and high (purple) effective reproductive numbers, for a different percent of non-severely symptomatic infections who seek care (5%, 25%, 50%, 75%, 100%). The solid line indicates the mean estimate under expanded antibiotic treatment guidelines, the shaded region represents the 25% and 75% quantiles, and the dashed line shows the final size of the outbreak under current antibiotic treatment guidelines (treating no non-severely symptomatic infections).
